# Content of Trans Fatty Acids in Human Cheek Epithelium: Comparison with Serum and Adipose Tissue

**DOI:** 10.1155/2013/276174

**Published:** 2013-10-08

**Authors:** Ransi A. Abraham, Vinay K. Bahl, Rajinder Parshad, V. Seenu, Ambuj Roy, Smita Golandaz, Prabhakaran Dorairaj, Lakshmy Ramakrishnan

**Affiliations:** ^1^Department of Cardiac Biochemistry, All India Institute of Medical Sciences, New Delhi 110029, India; ^2^Department of Cardiology, All India Institute of Medical Sciences, New Delhi 110029, India; ^3^Department of Surgery, All India Institute of Medical Sciences, New Delhi 110029, India; ^4^Centre for Chronic Disease Control, New Delhi, India

## Abstract

Studies pertaining to trans fatty acids (TFA), which have been implicated in development of chronic diseases, are more relevant in developing countries where nutrition transition is changing traditional habits and practices. Measuring TFA is an arduous task because of the need for fat biopsies. This study identifies a tissue, which can be easily accessed for analytical measurement of trans fatty acid. In this cross-sectional study, fatty acid in adipose tissue, cheek epithelium, and blood samples were assessed by gas chromatography. Spearman correlation coefficient was computed to study the correlation of fatty acid distribution among the three tissues. The correlation coefficient of total trans fatty acid between cheek epithelium and serum was 0.30 (*P* < 0.02) and between cheek epithelium and adipose tissue was 0.33 (*P* < 0.019). This study is the first to report trans fatty acid profile in cheek epithelium giving scope for utilizing the cheek epithelium as a tissue for objective assessment of trans fatty acid intake.

## 1. Introduction

Studies from developed countries have documented that the fatty acid content of the diet is an important determinant of risk of many chronic diseases, including arteriosclerosis, coronary heart diseases and that of essential fatty acid deficiencies [1]. The number and position of double bonds influence the function and metabolism of fatty acids; therefore, changes in the fatty acid composition of diets may have profound effects on several physiological processes [2]. Trans fats are unsaturated fatty acids with at least one double bond in the trans configuration and they occur in small amounts in some foods from animals; however, majority of the trans fatty acids are artificially created during partial hydrogenation. The main concern of their health effects is due to their structural similarity to saturated fatty acid, lack of specific metabolic functions, and competition with essential fatty acids [2]. 

A high trans fatty acid content has been reported in Indian fried snacks and sweets using secondary data [3]. Another recent study reported a high trans fatty acid content, 5–12 times higher than the standard set by WHO (2%) for reduced risk [4, 5]. In spite of reports of high intake of trans fatty acids among Indians, their role in the causation of chronic diseases is not well studied. The reasons for these are many and include the following. Lack of an objective biochemical measure of fat intake prevents the objective validation of an association between dietary fat and chronic diseases. Population studies exploring effects of trans fatty acid on disease rely on information of dietary intake gathered through food frequency questionnaires, which may not accurately reflect trans fat intake. The dietary estimates from self-reporting method also depend on food composition tables which may not adequately reflect recent dietary changes. Though analytical measures may be more reliable under such situations the gold standard biomarker, which is measurement of adipose levels of trans fatty acids, is difficult, as it entails obtaining fat biopsies or fat aspirates which are invasive in nature and thus of limited usage [6–8]. Blood fractions, serum, plasma, and erythrocytes are widely used in epidemiological studies to assess trans fatty acid intake but indicate short-term intake as compared to adipose tissue. Therefore, fatty acid pattern of cheek cell epithelium makes an attractive noninvasive alternative which has not been explored for trans fatty acid assessment.

Collection of cheek epithelium is noninvasive and can be done without trained clinical personal and independent of the location of a laboratory. Another advantage is the willingness of individuals to donate cheek cells rather than blood. The fatty acid levels assessed in cheek cell epithelium are not dependent on the postprandial state of subjects making it an attractive option in infants and children in whom collecting fasting sample is difficult [9]. Studies have shown that the polyunsaturated fatty acid (PUFA) profile of cheek cell phospholipids correlates significantly with that of plasma and (red blood corpuscles) RBC phospholipids [10–12]. We studied the cheek trans fatty acid level and correlated the same with that in adipose and serum.

## 2. Methods 

A cross-sectional study was carried out to meet the objective. 50 healthy subjects of 20–60 years of age undergoing elective abdominal surgery were recruited. Subjects who had a previous history of coronary artery disease, cancer, and endocrine disorder were excluded. Subjects who had gained or lost 5 kg weight in the previous two years and were on changed diet due to clinical condition in the same duration were also excluded. Written informed consent was obtained from all subjects. The study was approved by Ethics committee of the All India Institute of Medical Sciences, New Delhi. Height, weight, waist circumference, and hip circumference of all subjects were measured using standardized equipments. Information on dietary intake was collected by administering the dietary history questionnaire [13]. 10 mL blood was collected from subjects with overnight fasting, centrifuged, serum was separated and stored at −70°C till analysis. The cheek epithelium was collected on the morning of the surgery from subjects with overnight fasting. For collection of cheek cells, the patients were asked to rinse their mouth thrice with water to remove any food debris. The interior of the cheek was scrapped using sterile wooden spatula and was transferred to 50 mL falcon tube containing 5 mL distilled water. The tube was centrifuged, the cheek epithelium pellet was weighed, and 1 mg was transferred to an amber coloured vial containing a mixture of hexane : isopropanol (3 : 2 v/v) and stored at −70°C till analysis. Adipose was collected from subjects during the surgery, washed in saline to remove blood, stored in a amber colored vial containing a mixture of hexane : isoproponol (3 : 2 v/v), and kept at −70°C. BHT (Butylated Hydroxytoluene) as an antioxidant was added to all the samples before storage.

## 3. Fatty Acid Estimation

The lipids from cheek epithelium, adipose tissue, and serum were isolated by Folch method [14]. Briefly, cheek epithelium and adipose were sonicated for 30 seconds for disrupting the cells and lipids were extracted with the solvent chloroform : methanol (2 : 1 v/v). The extract was then transferred to 15 mL stoppered cultured tubes and dispersed in the solvent 20 times the original volume of the tissue sample. 200 *μ*L serum was similarly taken in 20 times volume of lipid extraction solvent in 15 mL stoppered culture tubes. After dispersion, the mixture was agitated for 2 hours on an orbital shaker at room temperature [15]. The solvent was washed with 0.2 volume of 0.9% NaCl solution. The upper phase was siphoned off and the lower chloroform phase containing lipids was evaporated under the stream of nitrogen. 

The extracted lipids were esterified using 500 *μ*L methylating reagent containing methanol-acetyl chloride 20 : 1 (v/v) by heating in water bath at 50°C for one hour. The esterified lipids were washed with 0.2 volume of 0.9% NaCl and methyl esters were extracted into hexane, evaporated under the stream of nitrogen, and stored in amber coloured vials till subjected to gas chromatography [16]. Extraction and methylation for cheek epithelium, adipose, and serum were performed in a same batch to eliminate batch to batch variability. The esters were redissolved in isooctane, C17:0 was added as an internal standard, mixed, and 1 *μ*L sample was taken in Hamilton syringe and injected into the fused silica capillary cis/trans column SP 2560, 100 m × 250 *μ*m internal diameters × 0.20 *μ*m film (Supelco, Belefonte, PA) attached to the gas chromatography (GC) instrument. Nitrogen gas was used as a carrier gas. The port temperatures of both the injector and the detector were set at 250°C. The oven temperature was initially set at 90°C for 4 min and was then increased 15°C/min until a temperature of 150°C was reached and held for 10 min; the temperature was further increased at 1°C/min till 170°C after which rate of temperature change was 5°C/min until a temperature of 230°C was reached and maintained for 30 minutes. A split ratio of 1 : 10 and an injection volume of 1 *μ*L were used. The separated fatty acid methyl esters were detected by Flame-ionization detector. Fatty acid methyl ester peaks were identified by comparing their retention times with those of known standards both individual as well as mixed (Fatty Acid Methyl Esters from SUPELCO). Each fatty acid peak in the unknown samples was identified with reference to C17:0 as well as by comparing with known standards. Peaks were quantified by calculating the area under the peak using software from AIMIL (Nucon Technologies). 

The fatty acids were also recalculated and grouped together in different categories as per their degree of unsaturation. The fatty acids included among total saturated fatty acids (TSFA) are C12:0, C14:0, C15:0, C16:0, and C18:0, and those in total monounsaturated fatty acids (TMUFA) are cis C14:1 cis, C16:1 cis, and C18:1 cis. Total polyunsaturated fatty acids (TPUFA) (cis) include C18:2 cis, cis, C18:3n-6, and C18:3n-3. Total trans fatty acid (TTFA) comprises of C16:1 9-trans, C18:1 9-trans and C18:1 11-trans, C18:2 trans, trans, C18:2 trans, cis and C18:2 cis, C18:3 trans trans trans, C18:3 trans trans cis, C18:3 trans cis cis, and C18:3 cis cis trans, C18:3 trans cis trans, C18:3 cis trans cis, C18:3 cis trans trans. Total long chain fatty acid (TLCFA) comprises of cis C20:4, cis C20:5, and cis C22:6.

## 4. Statistical Analysis

The fatty acids assessed in cheek epithelium, adipose tissue, and plasma were expressed as percentage of total fatty acid measured. Since the distribution of fatty acids was not normal median and interquartile range was computed in the three biological samples, monounsaturated, polyunsaturated, saturated, trans fatty acids, and long chain fatty acid total were recalculated from the sums of fatty acids detected in each category. Mann whitney *U*-test was performed to compare the fatty acids in the tissue pairs. Nonparametric spearman correlation coefficient was computed to study the correlation of fatty acid distribution in the three tissues. Correlation coefficients were considered significant at a level of *P* < 0.05. All data were analyzed using SPSS version 16.0 software (Statistical Product and Service Solutions, Chicago, IL, USA). 

## 5. Results

The baseline characteristics of the participants are described in Table 1. The mean age ± SD (Standard Deviation) of the subjects was 39 (8.8) with the mean (Body Mass Index) BMI of 23.7 (9.5) kg/m^2^. Majority of the subjects used mustard or rapeseed oil (86%) followed by sunflower oil (8%) and soybean oil (6%) for cooking. The frequency of eating fried food at home (46%) or outside (50%) was less than once per week. Eighty percent of the subjects reported eating bakery products more than 3 days a week, primarily biscuits and namkeens (salted savories). Majority of (70%) the subjects consumed milk on a daily basis. Even though 52% of the subjects were non vegetarians their frequency of intake was only once a week and comprised of mainly egg, chicken or goat meat; 38% consumed fish once a week. The intraday coefficient of variation (CV) of trans fatty acid (TFA) measurement was 6.55% and inter day CV was 13.25%. Duplicate runs of cheek epithelium showed an intraclass correlation (ICC) of 0.94 for total trans fatty acid measurement.

**Figure 1:**
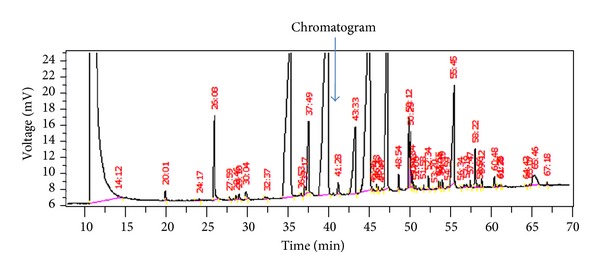
Chromatogram showing the fatty acid profile in a representative Cheek Epithelium sample. The numbers above the peaks are the retention time. The first peak is the solvent peak. The arrow shows the C:17 peak which was added as internal standard. The trans fatty acid peaks occur at the following retention times in this sample: 16:1t (36.53), 18:1t (43.33), 18:2 (45.46, 46.23, 46.43), and 18:3t (48.54, 50.33, 52.34, 53.30, 54.04, 54.59), respectively.

**Figure 2:**
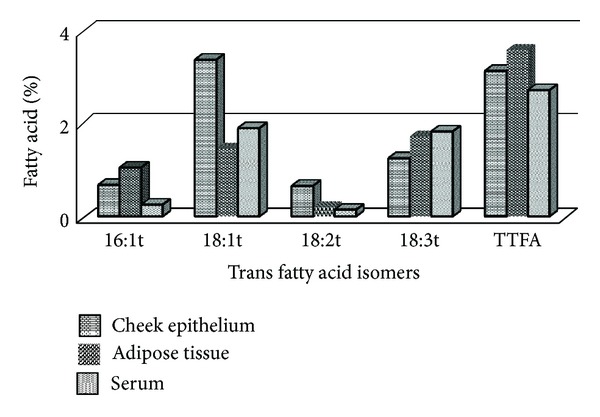
Distribution of trans fatty acid isomers in different tissues.

**Figure 3:**
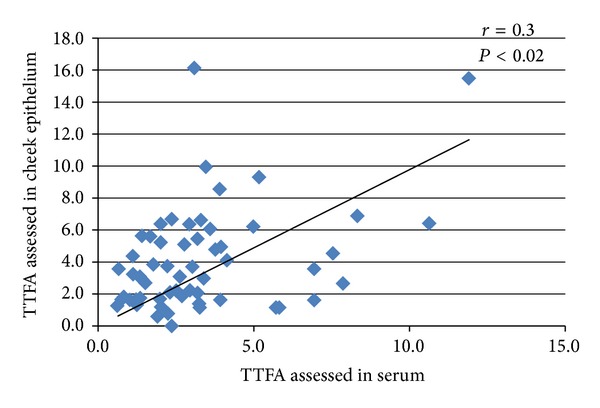
Scatter plot of total trans fatty acid in cheek epithelium and serum.

**Figure 4:**
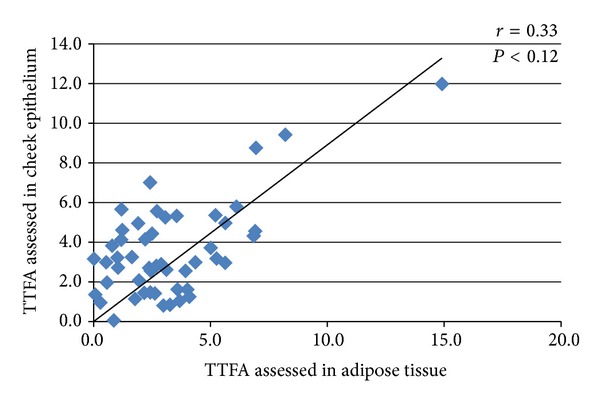
Scatter plot of total trans fatty acid in cheek epithelium and adipose tissue.

A representative chromatogram showing the fatty acid profile in cheek epitheliumis shown in Figure 1. The distribution of fatty acids in cheek epithelium, adipose tissue, and plasma are described as percentage of total fatty acid measured, expressed as median and intraquartile range in Table 2. Saturated fatty acid was the most abundant fatty acid across all three tissues. Adipose tissue and serum were richer in cis polyunsaturated fatty acid, 20% in both. The distribution of trans fatty acid was comparable in cheek epithelium, adipose, and serum with values of 3.10%, 3.56%, and 2.7%, respectively.  Figure 2 shows the distribution of trans fatty acid isomers in the tissues. Transisomer of palmitic acid (16:1t) was higher in adipose 1.04 [0.3,4.12] with the lowest in serum 0.25 [0.16, 0.59]. Transisomer of oleic acid (18:1t) was higher in cheek epithelium and comprised primarily of 18:1 11t (vaccenic acid). Transisomer of *α*-linolenic acid (18:3t) was also present in substantial amount, whereas the transisomer of linoleic acid (18:2t) was present in low levels. Correlation coefficient of total trans fatty acids assessed in cheek epithelium and serum was 0.30 (*P* < 0.02). Correlation coefficients were 0.66 (*P* < 0.0001) for total saturated fatty acid and 0.33 (*P* < 0.01) for total monounsaturated fatty acid. Comparison of cheek epithelium and adipose tissue showed significant correlation for TTFA (*r* = 0.33,   *P* < 0.019), TSFA (*r* = 0.35, *P* < 0.012), and TMUFA (*r* = 0.30, *P* < 0.03).  Figure 3 shows the scatter plot for total trans fatty acids in cheek and serum and Figure 4 that in cheek and adipose tissue. Among the individual trans fatty acids 18:1 trans in cheek significantly correlated (*r* = 0.51) with that in serum (*P* < 0.004).

## 6. Discussions

Trans fatty acid levels in cheek epithelium correlated significantly with that in serum and adipose tissue. Although studies have reported fatty acid assessment in cheek epithelium, there are no studies reporting trans fatty acid levels. McMurchie et al. reported measurement of cheek cell phospholipid fatty acid profile in subjects with different dietary fat intake and reported that the differences in dietary fat intake were closely reflected in the cheek cell phospholipids fatty acid profile [17]. In another study, the same authors observed changes in the proportion of linoleic acid in cheek if the dietary polyunsaturated fatty acid to saturated ratio was changed [18]. Cheek cell phospholipids fatty acids were found to reflect dietary intake in a study of preterm infants who were given n-3 long chain polyunsaturated fatty acid supplementation [12]. In another supplementation trial EPA and DHA in buccal cell correlated moderately well and significantly with EPA and DHA in the other sample types such as RBCs, plasma fraction, platelet, and adipose tissue assessed. An overall dose response in buccal cells was observed; however, there was no linear dose trend for EPA and DHA in the 12th month, suggesting that this pool may not adequately reflect long-term FA intake [19]. The fatty acid profile of cheek cell phospholipid reflected that of liver, skeletal muscle, and adipose tissue with reasonable degree of confidence in a study done in piglets fed experimental diet [20]. The fatty acid profile of cheek cell reported by Klingler et al. was similar to the profile in our study with palmitic acid, stearic acid, oleic acid, linoleic acid, and palmitoleic acids presenting the major fatty acids [21]. These studies were interventional in nature and did not report the trans fatty acid levels. The TSFA, TMUFA, and TTFA in cheek epithelium correlated significantly with serum and adipose tissue in our study. Among the individual trans fats, transisomer of palmitoleic acid was higher in adipose, a storage depot, than serum and cheek. The transisomer of palmitoleate (trans-16:1n-7) represents a distinctly exogenous source of 16:1n-7, unconfounded by endogenous synthesis or its determinants [22]. It is principally derived from naturally occurring dairy/ruminant trans fats [23] and is not associated with higher cardiovascular risk [24]. Transisomer of oleic acid identified in our study was primarily vaccenic acid 18:1 11t. Elaidic acid was present in only lesser amount. Transisomer of *α*-linolenic acid was present in substantial amount in all the tissues. It is introduced into oil during deodorization [25] and its levels are a reflection of the quality of oil used by the population. 

Adipose tissue has been widely accepted as a tissue of choice to study trans fatty acid as they reflect long-term storage while serum reflects short-term changes; however, obtaining the samples is difficult as it requires a painful invasive procedures. Cheek cell epithelium offers an attractive noninvasive option especially in interventional studies that requires repeated sampling. 

The correlation coefficient between buccal cell epithelium and serum for TSFA (0.66), TMUFA (0.33), and TPUFA (0.44) observed in our study seems to authenticate the assumptions that major origin of fatty acid in cheek is serum as they interact directly with the bloodstream across easily permeable barriers and hence show changes in diet more quickly than those tissues that are nurtured by diffusion through highly selective barriers [26, 27]. The content of 18:1t, 18:3t, and total TFA assessed in cheek epithelium, adipose tissue, and serum did not differ significantly. A significant correlation coefficient though weak was observed between tissue pairs. The reason for weak correlations observed could be due to the fact that tissues may vary in the endogenous synthesis of some fatty acids [28]. Nondietary factors such as genetic variation, disease status, lifestyle differences, apolipoprotein levels, and hormonal status influence deposition and mobilization of individual fat [29]. Baylin et al. have reported that the tissue fatty acid which are of exogenous source such as linoleic acid, *α*-linolenic acid, and 18:2 trans fatty acid show high correlation with diet in all tissues, but the distribution of fatty acids in different tissues varies due to metabolic changes and different physiological roles [30]. This is consistent with the correlations for saturated fatty acid and linoleic acid observed between serum and cheek epithelium in our study. Correlations may also be affected by differences in bioavailability or selective retention of fatty acids in certain tissue lipids. 

The adipose tissue and erythrocyte which reflect long-term or medium-term intake may not be suitable in studies such as clinical trials and community intervention studies that reports short-term changes. Moreover dietary intake assessment based on nutrient database is not foolproof as nutrient database, when available may not adequately reflect recent changes in food composition especially in developing countries where nutritional transition is occurring. Utilizing cheek cell epithelium, a noninvasive tissue, for assessing trans fatty acid profile in studies where a objective measurement is essential, instead of adipose tissue or serum seems a viable option.

Low yield of cheek cells in some subjects is a potential drawback of using it for fatty acid profiling in studies. However, a recent method published for the analysis of cheek cell glycerophospholipid fatty acid may overcome these limitations [31]. We did not look at the different fractions, cholesterol ester, phospholipids, and triglyceride in our study. However, experimental studies have shown that all three fractions respond similarly to short-term increases in dietary intake [30, 32–34] which we hope to capture in a noninvasive tissue such as cheek cell. Hence, trans fatty acid assessment in cheek epithelium makes an attractive noninvasive option. The study could be carried out only in smaller number of subjects due to the difficulty in obtaining adipose tissue samples.

## 7. Conclusion

Cheek cell epithelium has been gaining prominence as a sample to study various biological characteristics in individuals. The use of cheek epithelium in the analysis of trans fatty acid is of potential use in dietary and nutritional studies in human subjects especially children as they can be obtained in a completely noninvasive manner. The study demonstrates the potential suitability of cheek cell epithelium, a completely noninvasive tissue for objective assessment of trans fatty acid particularly where large population level assessment of trans fatty acids are needed.

## Figures and Tables

**Table 1:** The baseline characteristics of the subjects.

Variable	Mean ± SD
Age (yrs)	39 ± 8.8
BMI (Kg/m^2^)	23.7 ± 9.5
Waist circumference (cm)	88.86 ± 19.7
Hip circumference (cm)	95.94 ± 18.33
Systolic blood pressure (mmHg)	120 ± 10.6
Diastolic blood pressure (mmHg)	79 ± 7.59

SD: standard deviation.

BMI: body mass index.

**Table 2:** Distribution of fatty acids (median (interquartile range)) in cheek epithelium, adipose tissue, and serum.

Fatty acid	Cheek epithelium	Adipose tissue	Serum
C12:0	0.47 (0.23, 0.77)	0.44 (0.33, 0.64)	0.29 (0.11, 0.49)
C14:0	3.2 (2.6, 3.63)	4.05 (3.38, 5.43)	1.76 (1.36, 2.69)
C15:0	0.39 (0.26, 0.59)	0.35 (0.15, 0.51)	0.25 (0.17, 0.31)
C16:0	37.25 (30.87, 42.44)	36.28 (29.58, 44.04)	32.19 (28.14, 36.65)
C18:0	20.78 (14.44, 28.06)	4.37 (2.5, 10.86)	11.9 (8.06, 16.96)
TSFA	62.02 (51.35, 74.49)	46.64 (39.47, 61.28)	47.21 (38.06, 57.64)
C14:1	0.38 (0.21, 0.86)	0.4 (0.19, 0.88)	0.19 (0.11, 0.44)
C16:1	1.52 (0.64, 2.33)	0.45 (0.18, 2.81)	2.17 (1.21, 2.96)
C18:1	19.95 (8.82, 27.2)	17.18 (9.87, 29.84)	21.03 (13.68, 24.21)
TMUFA	21.44 (11.53, 29.46)	21.95 (13.71, 32.39)	23.25 (13.98, 26.89)
C16:1t	0.67 (0.27, 1.31)	1.04 (0.3, 4.12)	0.25 (0.16, 0.59)
C18:1t	3.34 (1.61, 4.12)	1.45 (0.62, 2.41)	1.88 (1.17, 2.24)
C18:2t	0.65 (0.16, 1.42)	0.2 (0.05, 0.48)	0.14 (0.07, 0.39)
C18:3t	1.24 (0.88, 2.66)	1.71 (1.06, 2.52)	1.81 (1.08, 2.55)
TTFA	3.10 (1.66, 5.58)	3.55 (1.93, 6.73)	2.69 (1.79, 3.58)
C18:2	5.07 (2.4, 10.39)	17.66 (12.82, 21.46)	19.93 (14.62, 25.04)
C18:3n3	0.33 (0.17, 0.82)	0.23 (0.1, 0.48)	0.37 (0.2, 0.58)
C18:3n6	0.98 (0.41, 3.3)	1.29 (0.53, 2.48)	0.54 (0.16, 1.39)
TPUFA	8.07 (6.11, 11.44)	20.07 (13.95, 23.71)	21.16 (16.19, 26.42)
C20:4	2.44 (0.82, 3.54)	1.03 (0.36, 3.17)	5.27 (4.18, 6.32)
C20:5	0.33 (0.12, 1.37)	0.1 (0.05, 0.17)	0.45 (0.21, 0.88)
C22:6	0.63 (0.25, 0.92)	0.07 (0.05, 0.14)	0.68 (0.44,0.93)
TLCFA	3.04 (1.92, 4.34)	1.21 (0.43, 3.35)	5.75 (4.68, 7.31)

Fatty acid are expressed as % of total fatty acid.

TSFA: total saturated fatty acid; TMUFA: total monounsaturated fatty acid; TTFA: total trans fatty acid; TPUFA: total polyunsaturated fatty acid; TLCFA: total long chain fatty acid.

## Abbreviations

BHT: Butylated hydroxytoluene
BMI: Body mass index
C: Carbon
CAD: Coronary artery disease
GC: Gas chromatography
ICC: Intraclass correlation
PUFA: Polyunsaturated fatty acid
RBC: Red blood corpuscles
TFA: Trans fatty acid
TMUFA: Total monounsaturated fatty acid
TLCFA: Total long chain fatty acid
TPUFA: Total polyunsaturated fatty acid
TSFA: Total saturated fatty acid
TTFA: Total trans fatty acid
SD: Standard deviation
SPSS: Statistical product and service solutions.
